# Laboratory diagnosis of pertussis: A survey on provincial public health laboratory methods

**DOI:** 10.14745/ccdr.v52i05a03

**Published:** 2026-05-31

**Authors:** Courtney Meilleur, Jennifer Grant, Gregory Tyrrell, Jessica Minion, Paul Van Caeseele, Julianne Kus, Brigitte Lefebvre, Todd Hatchette, Guillaume Desnoyers, Lei Jiao, Heidi Paulin, Raymond Tsang

**Affiliations:** 1 Vaccine Preventable Bacterial Diseases, National Microbiology Laboratory, Public Health Agency of Canada, Winnipeg, MB; 2 British Columbia Centre for Disease Control, Vancouver, BC; 3 Provincial Laboratory for Public Health, Edmonton, AB; 4 Roy Romanow Provincial Laboratory, Regina, SK; 5 Cadham Provincial Laboratory, Winnipeg, MB; 6 Public Health Ontario Laboratory, Toronto, ON; 7 Laboratoire de santé publique du Québec, Institut nationale de santé publique du Québec, Sainte-Anne-de- Bellevue, QC; 8 Pathology and Laboratory Medicine Program, Nova Scotia Health Authority, Halifax, NS; 9 New Brunswick Public Health Laboratory, Dr. Georges-L.-Dumont University Hospital Centre, Moncton, NB; 10 Newfoundland and Labrador Public Health Laboratory, St. John’s, NL; 11 Provincial Laboratory Services, Health Prince Edward Island, Charlottetown, PE

**Keywords:** pertussis, national surveillance program, recommendations, diagnostic procedures, strain characterization

## Abstract

**Background:**

Pertussis, a vaccine preventable respiratory illness caused by the bacterium *Bordetella pertussis* (*B. pertussis*), has been a nationally reportable disease in Canada for over 100 years; however, cases resurged in Canada and globally in 2023–2024.

**Objective:**

To examine the breadth and depth of pertussis strain surveillance currently being carried out across Canada.

**Methods:**

A survey was sent to all ten provincial public health laboratories inquiring how pertussis was diagnosed or identified in the laboratory, including the polymerase chain reaction (PCR) diagnostic methods, bacteriological culture, identification and strain characterization such as molecular typing and antibiotic susceptibility testing.

**Results:**

Nine of the ten provincial laboratories provided responses. Five provincial laboratories reported performing bacteriological culture, and some only from specimens that tested positive by PCR. Long-term storage of submitted and historical specimens took place in six laboratories. Identification of *B. pertussis* was commonly done through matrix-assisted laser desorption/ionization time-of-flight mass spectrometry (MALDI-TOF MS) analysis, though immunochemical and PCR-based methods were also used. One laboratory conducted antibiotic susceptibility testing in specific circumstances. No laboratory performed fimbriae serotyping or examined expression of other pertussis vaccine antigens. One laboratory used whole-genome sequencing for outbreak investigation. The PCR diagnostics were performed in eight of the responding laboratories and always include IS481 and pIS1001 gene targets. Some laboratories also reported using other gene targets to identify and distinguish between *B. pertussis*, *B. parapertussis*, *B. holmesii* and *B. bronchiseptica.*

**Conclusion:**

Given the global increase in pertussis, with the emergence of macrolide-resistant and pertactin-deficient strains, strain characterization should be added to the Canadian national pertussis surveillance program.

## Introduction

Pertussis, which is commonly referred to as whooping cough, is caused by the bacterium *Bordetella pertussis* (*B. pertussis*) (1). Pertussis has been a notifiable disease in Canada since 1924 (2). Surveillance of pertussis is done by provinces and territories and they in turn voluntarily report cases to the Public Health Agency of Canada (PHAC)’s Canadian Notifiable Disease Surveillance System. Data collected and validated by the Canadian Notifiable Disease Surveillance System are published annually online in the Notifiable Diseases Online website (3). The PHAC also supports the Canadian Immunization Program ACTive (IMPACT) to carry out hospital-based surveillance of paediatric pertussis cases ((4)).

For control of pertussis, an inactivated whole-cell vaccine was introduced in Canada in 1943, which was subsequently replaced by the adsorbed whole-cell vaccine from 1981 to 1985. Eventually, acellular pertussis vaccine was introduced in 1997 because of its less reactogenic nature ((5)). Despite having a vaccine, pertussis continues to cause disease in infants, young children, adolescents and adults for various reasons related to vaccine hesitancy, waning of vaccine-induced protective immunity and divergence of current circulating and vaccine strains ((6)).

Between 2020 and 2022, COVID-19 pandemic restrictions caused disruptions in social gatherings, which resulted in lower numbers of different respiratory infections, including pertussis ((7)). As with other respiratory infections ((8,9)), pertussis appears to have re-emerged after the pandemic to cause more cases than before the pandemic ((10,11)). Data from 29 European Economic Area countries reported 1,578 and 2,623 cases of pertussis in 2021 and 2022, respectively ((12,13)). In 2023, the number of pertussis cases reported by these countries jumped to over 25,000 cases; and in the first three months of 2024, more than 32,000 cases were reported ((14)). In China, pertussis increased in 2022 and 2023 with 39,781 and 38,205 cases reported, respectively ((15)). In the first two months of 2024, 32,380 cases, including 13 deaths, were recorded ((16)). Furthermore, the disease has shifted from mainly affecting infants to older children, and a new strain showing macrolide resistance and pertactin deficiency emerged ((17)). In South Korea, a national epidemic was described in 2024 with the highest incidence rate found in those aged 13 years, with 526.2 cases per 100,000 population ((18). In addition, the macrolide-resistant strain appeared to have spread to some Asian countries ((19). Besides monitoring for susceptibility to macrolides, the European Centre for Disease Prevention and Control (ECDC) also recommends performing serotyping, multi-locus antigen sequence typing and vaccine antigen expression by enzyme-linked immunosorbent assay (ELISA) or gene sequencing ((20). Similar strain surveillance programs are also available at the United States (US) Centers for Disease Control and Prevention and the United Kingdom Health Security Agency.

In 2024, the Pan American Health Organization also reported increases in pertussis in multiple countries within the Americas including the US, Brazil, Mexico and Peru ((21). For example, preliminary data in the US show that reported cases of pertussis in 2024 increased six-fold when compared to 2023 ((22)). Canada was no different from other nations, reporting increases in pertussis cases in multiple jurisdictions leading to significant case numbers not seen since prior to the introduction of the pertussis vaccines. The increases in pertussis cases occurred in nearly all provinces and territories ((23–29)) (*personal communication Dr. Paul Van Caeseele, March 31, 2025*). For the first eleven months in 2024, Public Health Ontario reported 1,634 pertussis cases, with 1,396 as confirmed and 239 probable cases. This led to the highest incidence rates since 2017 in those younger than one year old and those between the ages of 10 and 14 years old (74.2 and 55.2 per 100,000, respectively). According to Québec public health, 16,130 cases of whooping cough were recorded January 1–October 9, 2024. In Newfoundland and Labrador, 230 confirmed cases of pertussis were recorded throughout the province by September 10, 2024.

Given the global resurgence of pertussis after the COVID-19 pandemic, with the emergence of macrolide-resistant and vaccine antigen-deficient strains ((30,31), the capacity in Canada for a national surveillance program that includes strain characterizations and antibiotic susceptibility testing must be re-examined. It is with this understanding that the National Microbiology Laboratory Branch of the PHAC is working with provincial and territorial public health laboratory partners to examine how pertussis is diagnosed and characterized in Canada in order to understand the breadth and depth of pertussis surveillance in the country.

## Methods

This study was intended to obtain details on strain characterization work, which can contribute to the Canadian national surveillance of pertussis. Strain characterizations are often done at the provincial public health laboratories, which also serve as reference laboratories for their provinces as well as neighboring territorial governments. The PHAC’s National Microbiology Laboratory Branch has a close working relationship with the public health agencies in all provinces and territories as partners in public health microbiology issues. This relationship is formalized as a national association of public health laboratory professionals, established in 2001 as the Canadian Public Health Laboratory Network, with a role to provide rapid, coordinated and unified laboratory response to emerging and re-emerging infectious diseases ((32). As such, frontline and hospital laboratories as targets of the survey were not included.

Therefore, on November 17, 2024, an e-mail was sent to the medical microbiologists or medical directors of ten provincial public health laboratories explaining the purpose of the survey and a questionnaire with questions covering bacteriological culture of *B. pertussis*, polymerase chain reaction (PCR) diagnostic method and strain characterization. For strain characterization, the following questions were asked: 1) how long are cultures preserved; 2) how cultures are identified and characterized including serotyping (for expression of fimbriae antigens); 3) expression of other vaccine antigens; 4) molecular typing, and 5) antibiotic susceptibility testing. For PCR diagnostic methods, details of the method used (commercial kit/platform or laboratory developed method), the gene targets detected in the PCR assays and positivity cut-off values were included in the questionnaire. A copy of the survey questionnaire can be found in the **Appendix** and participants had up to December 31, 2024 to respond voluntarily.

Since the survey did not request any personal information and was done within the autonomy of the Canadian Public Health Laboratory Network for public health purposes, institutional research ethics approval was not sought nor was informed consent necessary as the response was voluntary. Data protection was only applied with laboratories identified by numbers instead of naming the laboratory linked to the data captured in the survey. The survey questions were based on questions received by the National Microbiology Laboratory Branch from public health practitioners across the country, such as antibiotic resistance patterns and strain types found.

## Results

Responses to the questionnaire were received from nine of the ten provincial public health laboratories. One provincial public health laboratory did not respond to the survey.

Culture of *Bordetella pertussis* and subsequent testing on *Bordetella pertussis* isolates

Five provincial public health laboratories performed bacteriological culture of primary specimens for isolation of *B. pertussis*, although one of them reported doing so only rarely in recent years. Three of these five provincial laboratories disclosed that only specimens tested positive by PCR for *B. pertussis* were subjected to culture, including one of these laboratories only performed culture on specimens that were PCR-positive with low cycle threshold (Ct) values. Six provincial laboratories reported long term (on the scale of years) storage of *B. pertussis* isolates; although some laboratories had stopped performing primary cultures, historical isolates were preserved and stored. Two provincial laboratories also reported storage of primary specimens submitted for *B. pertussis* testing at −80°C.

Among those performing primary bacteriological culture and/or performing bacterial identification as a provincial public health reference laboratory, six laboratories (including one laboratory that did not provide primary culture service but received samples for reference diagnostic identification testing) identified *B. pertussis* by matrix-assisted laser desorption/ionization time-of-flight (MALDI-TOF). One of these six laboratories also used biochemical tests such as oxidase, motility, growth on MacConkey agar, and genetic sequencing for identification of *B. pertussis* and other uncommon *Bordetella* species, while another laboratory also used bacterial agglutination and fluorescent antibody tests for identification of *B. pertussis* and *B. parapertussis*.

Only one laboratory reported performing antibiotic susceptibility testing for *B. pertussis* specimens when requested by clinicians. No laboratory was reporting any phenotypic or genetic typing, including looking at expression of vaccine antigens or sequencing of vaccine antigen genes. Only one laboratory reported performing whole-genome sequencing on *B. pertussis* and *B. parapertussis* for outbreak investigation. The scope of bacteriological culture from primary specimens, and the subsequent testing to characterize the strains in the different provincial public health laboratories are summarized in **Table 1**.

**Table 1:** Primary culture of *Bordetella pertussis* from clinical specimens, identification method, antibiotic susceptibility testing and routine typing provided across provincial public health laboratories in Canada

Laboratories	Primary culture	Identification method	Antibiotic susceptibility testing	Routine typing for strain characterization	Culture preservation	Comments
1	Yes	MALDI-TOF plus PCR	No	No	Yes, years	-
2	Yes^a^	MALDI-TOF	Yes^b^ E-test	No	Yes, years	Whole-genome sequencing for outbreak investigation
3	Yes	MALDI-TOF	No	No	Yes, more than 10 years	-
4	Yes^c^	MALDI-TOF	No	No	Yes, years	-
5	Yes^d^	MALDI-TOF plus agglutination and FA^e^	No	No	Yes, years	-
6	No^f^	MALDI-TOF plus biochemical testing (16S rRNA sequencing if necessary)	No	No	Yes^g^, years	-
7	No	N/A	No	No	No	-
8	No	N/A	No	No	No	-
9	No	N/A	No	No	No	-
10	NA	N/A	N/A	N/A	N/A	No response

Abbreviations: FA, fluorescent antibody; MALDI-TOF, matrix-assisted laser desorption/ionization time-of-flight; N/A, not applicable; PCR, polymerase chain reaction; -, no comment

^a^ Only on PCR-positive samples

^b^ Only when requested by clinicians

^c^ Only when submitted on appropriate culture collection and transport media

^d^ Only on PCR-positive samples with low cycle threshold (Ct) values

^e^ Fluorescent Antibody Test

^f^ However, receives suspected *Bordetella* specimens as a provincial reference laboratory for identification

^g^ Received suspected *Bordetella* specimens for identification purpose

### Polymerase chain reaction diagnosis of pertussis

Three provincial public health laboratories used commercially available test kits or platforms for the laboratory diagnosis of pertussis: the R-Biopharm AG RIDA®GENE Bordetella ((33), the Diasorin Simplexa^TM^ Bordetella Direct Test ((34) and the QuidelOrtho Corporation’s Solana® Bordetella Complete Assay ((35). Five other provincial laboratories used laboratory developed tests (LDT), and while the gene targets detected by specific primer-probe sets in these LDTs may differ, they invariably included IS481 and pIS1001 (for the simultaneous detection and differentiation or identification of *B. pertussis* and *B. parapertussis*). Some laboratories also employed additional gene targets such as hIS1001 (for detection of *B. holmesii*), BHrecA (for detection of *B. holmesii*), bfrZ (for detection of *B. bronchiseptica*) and IS1002 (for detection of *B. parapertussis*). The gene targets used in the PCR assays by the various laboratories and their ability to identify and differentiate common *Bordetella* species are summarized in **Table 2**. Five laboratories that used LDTs also reported using Ct values to interpret the PCR results. Positive PCR results for pertussis were defined by Ct values ranging from less than or equal to 35 to less than 45 (Table 2).

**Table 2:** Polymerase chain reaction gene targets for molecular detection/diagnosis of *Bordetella pertussis* and other *Bordetella* species

Laboratories and PCR method	IS481	IS1001	IS1002	hIS1001 (*B. holmesii*)	bfrZ	BHrecA (*B. holmesii*)	Ct values for defining positive PCR results	Comments
1^a^	√	√	-	√	√	-	≤35	Can identify all four species including *B. bronchiseptica* with species-specific PCR; however, may not identify co-infection of Bp and Bh.
2^b^	√	√	-	√	-	-	N/A	Can identify Bp accurately most of the time; but may not identify co-infection of Bp and Bh; cannot differentiate between *B. para* and Bb.
3^a^	√	√	-	√	-	-	<45	Can identify Bp accurately most of the time; but may not identify co-infection of Bp and Bh; cannot differentiate between *B. para* and Bb.
4^c^	√	√	-	-	-	-	Built-in cut off values^d^	Can identify and differentiate between Bp and *B. para* accurately most of the time; however, *B. para* and Bb may be misidentified; also Bp and Bh may be misidentified.
5^a^	√	√	-	-	-	√	≤36	Method described in J Clin Microbiol 2010;48 (4):1435–7.
6	N/A	N/A	N/A	N/A	N/A	N/A	N/A	Not applicable or do not offer PCR diagnostic service at the provincial public health laboratory.
7^a^	√	√	-	√	-	-	40	Can identify Bp accurately most of the time; but may not identify co-infection of Bp and Bh; cannot differentiate between *B. para* and Bb.
8^a^	√	√	√	-	-	-	40	Can identify Bp and *B. para*; but in IS481+/IS1001−/IS1002− samples, differentiation between Bp and Bh/Bb is not possible (due to Bb may contain low copy numbers of IS481). In IS1001+/IS1002− samples, differentiation between *B. para* and Bb is not possible (due to Bb possibly containing low copy numbers of IS1001).
9^c^	√	√	-	-	-	-	Built-in cut off values^e^	Can identify and differentiate between Bp and *B. para* accurately most of the time; however, *B. para* and Bb may be misidentified; also Bp and Bh may be misidentified.
10	N/A	N/A	N/A	N/A	N/A	N/A	N/A	No response

Abbreviations: Bb, *Bordetella bronchiseptica*; Bh, *Bordetella holmesii*; Bp, *Bordetella pertussis*; *B. para*, *Bordetella parapertussis*; Ct value, cycle threshold value; N/A, not applicable; PCR, polymerase chain reaction; -, not tested in this laboratory

^a^ Laboratory-developed test

^b^ Commercial diagnostic test kit

^c^ Commercial diagnostic test platform

^d^ Approximately 37

^e^ Not given, measured and interpreted fluorescent signal

## Discussion

Nine of the ten provincial public health laboratories responded to this survey and the data obtained formed the basis of this report. Of the eight provincial public health laboratories that provide diagnostic PCR assays, five are able to detect *B. pertussis*, *B. parapertussis* and *B. holmesii*, and three were able to detect only *B. pertussis* and *B. parapertussis*. Thus, not all laboratories could definitively identify these three most important species by PCR. Bacteriological culture to recover viable *B. pertussis* from clinical specimens was only performed in five of the responding laboratories, and often only on PCR-positive clinical samples. The MALDI-TOF was the most common method used to identify *B. pertussis* cultures (used by all laboratories that provide this service of reference diagnostic/identification); however, only one laboratory carried out antibiotic susceptibility testing by E-test when requested by clinicians. Furthermore, none of the laboratories carried out routine typing of isolates to understand characteristics of the circulating *B. pertussis* strain. Therefore, current laboratory results do not contribute additional information towards the understanding of how strain characteristics may alter the epidemiology of pertussis in Canada.

At this time, the national surveillance of pertussis depends on provinces and territories reporting cases with minimal demographic information, including age and gender. The IMPACT surveillance of pertussis focuses on hospitalized cases with some additional clinical information ((4)). One deficient area is the lack of information on the *B. pertussis* bacteria currently circulating in Canada, which is needed to understand if the increase in Canadian pertussis cases in 2024 was due to expansion of a clonal strain or to diverse strain types. Resistance to oral macrolide antibiotics, such as erythromycin or clarithromycin, has been reported elsewhere and may also have emerged in circulating Canadian strains. It is with this understanding that the laboratory methods currently being used by our provincial partners for the surveillance of this highly contagious bacterial disease was reviewed.

Like for other common pathogens, strain characterization is an important component to an overall understanding of the evolving nature of the changing epidemiology of pertussis in Canada and globally. For example, once mainly a childhood disease, it now appears that depending on the locality, a noticeable proportion of cases occur either in older children, adolescent, adults or elderly. Several studies in the late 1990s and in early 2000s have described genetic polymorphisms in the vaccine antigen genes (e.g., pertactin and pertussis toxin) leading to the suggestion of divergence between current circulating *B. pertussis* strains and the strains used to manufacture vaccines ((36–40)). Genetic polymorphisms in the fim3 gene that encode fimbriae 3 or serotype 3 antigen have also been observed. In Canada, since the 1970s, the majority of *B. pertussis* isolates examined expressed the fimbriae 3 antigen but rarely the fimbriae 2 antigen. Additionally, non-synonymous mutations of the fim3 gene resulted in amino acid changes on a surface epitope of the fimbriae antigen led to the postulation that the polymorphisms may be subjected to immune pressure or selection from vaccine induced or naturally occurring immune response ((41)). Recent studies have also shown that genetic changes that favour the increase production of pertussis toxin (such as having the ptxP3 allele) have been associated with pertussis resurgence ((42)). Also, recent global *B. pertussis* isolates are frequently deficient in the expression of the pertactin antigen, including isolates in Canada ((43–48)). Furthermore, over a period of nine years, *B. pertussis* samples recovered from one province had the potential to fluctuate between different genotypes and expression of the vaccine antigen pertactin ((49)).

Although it is not fully understood 1) how these genetic polymorphisms within the vaccine antigen genes or 2) how strains not expressing certain vaccine antigens (such as Canadian isolates not expressing fimbriae 2 and pertactin antigens) would affect vaccine efficacy, it is expected that further antigenic drift away from the *B. pertussis* vaccine strains and their encoded antigens will negatively affect vaccine efficacy. Therefore, this highlights the importance of incorporating strain characterization into the overall pertussis surveillance program in Canada. Strain characterization may also mitigate potential emergence of antibiotic resistance, as in the case of a large increase in the number of pertussis cases due to the emergence of an erythromycin-resistant *B pertussis* strain in China ((50)).

In Canada, as in many other countries, most pertussis cases are diagnosed in the laboratory by PCR. This is likely because *B. pertussis* is a nutritionally fastidious bacteria that requires specialized and enriched culture media to support growth. The commonly used culture media for bacteriological isolation from primary specimens include both the Bordet-Gengou agar and the Regan-Lowe charcoal blood agar, which contain starch and/or charcoal to neutralize toxic fatty acids and peroxides, defibrinated horse or sheep blood, and sometimes antibiotics such as cephalexin to inhibit normal flora present in the nasopharynx. Also, bacteria can only be recovered during the first two weeks of cough, while PCR would remain positive for up to three or four weeks after disease onset. As such, bacterial culture has become less popular in frontline laboratories, which are increasingly adopting PCR assays that can simultaneously detect and identify a number of respiratory pathogens (e.g., BioFire Respiratory Panel [RP]2.1-EZ, which can detect up to 19 respiratory pathogens including *B. pertussis* and *B. parapertussis*). Also, the practice of PCR diagnostics for pertussis may varies by province (e.g., more reliance on provincial public health laboratory to provide this service in some provinces versus decentralized testing in others). Similarly, the ability to detect *Bordetella* species (e.g., *B. holmesii* and *B. bronchiseptica*) that may also cause cough symptoms varies across the country.

Not all the commercially available test kits and platforms or LDT for detection of pertussis by nucleic acid amplification testing (NAAT) can detect and differentiate between pertussis-causing *B. pertussis* and pertussis-symptoms like causing *B. parapertussis* and *B. holmesii*. To detect and differentiate these three *Bordetella* species, a NAAT needs to have specific primers and/or probes that target these three species separately ((51,52)). One such specific gene target for *B. pertussis* is ptxA. *Bordetella bronchiseptica* may also cause respiratory infections with cough in immunocompromised individuals ((53)). For detection and identification of *B. bronchiseptica*, yet another set of primers and/or probes would be required ((54,55)). The challenge in using NAAT with minimum numbers of primer pairs and probes is due to the fact that, for example, *B. holmesii* has been reported to harbor low copy numbers of IS481 ((56)) while *B. bronchiseptica* has been reported to harbor low copy numbers of IS481 and IS1001 ((57)). The IS481 and IS1001 are often used in PCR assays to detect *B. pertussis* and *B. parapertussis*, respectively.

While NAAT may be able to detect and differentiate or identify important *Bordetella* species involved in causing respiratory infections in human, the sensitive nature of this laboratory method may require some additional considerations in the interpretation of the test result. First, results from a NAAT for pertussis must be interpreted in the context of the clinical setting. For example, in Canada, a laboratory-confirmed case of pertussis is defined either by bacteriological isolation of *B. pertussis* or by detection of *B. pertussis* DNA from an appropriate clinical specimen, together with at least one compatible clinical findings of either cough lasting for two weeks or longer, paroxysmal cough of any duration, cough with inspiratory “whoop” or cough ending in vomiting or gagging, which may be associated with apnea ((58)). Secondly, contamination from the environment with the organism or its DNA may introduce potential false positive results. For example, pseudo-outbreaks of pertussis have been described in the literature ((59–61)). Therefore, the US Centers for Disease Control and Prevention also recommends that culture confirmation of at least one case should occur during the setting of a suspected pertussis outbreak ((62)).

In summary, maintaining *B. pertussis* culture capacity as well as strain identification and characterization including antibiotic susceptibility testing should remain available at either the provincial and/or national level pending further discussions on the most cost-effective surveillance program that meets the need of Canadian public health. Previously National Microbiology Laboratory has performed serotyping using monoclonal antibodies to *B. pertussis* fimbriae 2 and fimbriae 3, western immunoblot to detect the expression of vaccine antigen pertactin and genotyping of vaccine antigens genes, including pertussis toxin subunit A and pertussis toxin promoter region, fimbriae 3, pertactin and filamentous hemagglutinin ((41,43,49)).

In 2002, the Government of Canada organized a national consensus conference on pertussis with recommendations concerning laboratory diagnosis and surveillance (63). As a follow up, the National Microbiology Laboratory gathered Canadian experts on the subject of pertussis in a workshop to discuss the recommendations on laboratory diagnosis and surveillance from the national consensus conference. One of the action plans from this workshop was to set up a Working Group to discuss implementing a national strain characterization program ((64)). Due to competing priorities, this plan was put on hold, but in view of global resurgence of pertussis, the concern of antibiotic resistance and circulation of strains not expressing vaccine antigens, it may be time to re-examine this plan and put it into action.

### Limitations

Limitations of this study include not sending this survey to frontline laboratories (including private medical laboratories), hospital laboratories and regional public health laboratories. Therefore, important laboratories may have been missed that may be providing bacteriological cultures for the laboratory diagnosis of pertussis, and also the overall PCR technology being offered for detection of pertussis, including the popular molecular diagnostic platform like the BioFire Respiratory Panel [RP]2.1-EZ for simultaneous detection and identification of 19 respiratory viral and bacterial pathogens including *B. pertussis* and *B. parapertussis*. To reach out to all the frontline medical and hospital laboratories would have been a big undertaking. Nevertheless, six regional laboratories were contacted through one provincial public health laboratory to gather frontline information for comparison. None of these six responding laboratories reported providing bacteriological culture for pertussis; two laboratories use BioFire for the detection of *B. pertussis* and *B. parapertussis* (including one of these two laboratories also having an LDT) and one laboratory uses a LDT that targets only IS481. None of these six laboratories provided any testing to identify, type or determine antibiotic susceptibility for *B. pertussis*. This is likely because, at least in some provinces, testing for *B. pertussis* may be regarded as specialized testing and therefore perceived as best handled at the provincial public health reference laboratories.

## Conclusion

This survey has reviewed the current situation of pertussis in Canada and elsewhere globally, summarized the laboratory diagnostic procedures used in the Canadian Public Health Laboratory Network, identified some gaps in the national surveillance system and made recommendations to eliminate gaps identified. While PCR assays to detect *B. pertussis* is widely available across the country, culture capability may be more limited to some larger provinces. Routine strain typing that can inform strain characteristics such as expression of vaccine antigens, genetic polymorphisms that may affect a mismatch between the vaccine strains and current circulating strains and antimicrobial resistance data are currently lacking. Maintaining some bacteriological culture and strain characterization capability is essential for understanding effects of vaccine induced immune pressure on *B. pertussis*. A system to collect data representative of national distribution of strain types (including antimicrobial resistance) is essential to understand the evolving nature of pertussis and to prepare for potential need of newer vaccine strain. A sentinel surveillance system including collection of strain typing data coupled with epidemiological information is recommended for a comprehensive understanding of the current epidemiology of pertussis in Canada. Moving forward, strain collection and characterization with antibiotic susceptibility monitoring should be included in a sentinel surveillance system to understand the evolving nature of *B. pertussis* under national infant, adolescent and maternal pertussis immunization programs.

## Appendix

List 1: Questionnaire on laboratory diagnosis of *Bordetella pertussis* infection

1. Does your laboratory (province) perform bacterial culture of *Bordetella pertussis* and other *Bordetella* species? Yes ______; No ______

2. a) Describe the PCR method (and PCR targets) your laboratory (province) performs for detection of pertussis? _____________________

b) Is the assay able to differentiate between *B. pertussis*, *B. parapertussis*, and *B. holmesii*? _____________________

c) If your laboratory is performing qPCR: Yes ______; No ______; what is the Ct value used to determine a positive result (presence of pertussis)? ______

3. a) Subsequent to culture, what method do you use to identify it as *B. pertussis* and not other *Bordetella* species? _____________________

b) Does your laboratory (province) perform phenotypic and/or molecular typing: Yes ______; No ______

c) Does your laboratory test for antibiotic susceptibility? Yes ______; No ______

i. If yes, by what method: disk diffusion (); E-test (); micro-broth dilution method for MIC (); agar dilution method for MIC ().

d) How long do you keep your positive cultures? ______ Months; ______ Years
